# LONGITUDINAL PATTERNS OF COGNITION CHANGES AMONG KOREAN OLDER ADULTS WITH NO PARTNERS: A LATENT PROFILE ANALYSIS

**DOI:** 10.1093/geroni/igad104.2950

**Published:** 2023-12-21

**Authors:** Yeoju Kim, Giyeon Kim

**Affiliations:** Chung-Ang University, Seoul, Republic of Korea; Chung-Ang University, Seoul, Republic of Korea

## Abstract

Cognitive function decline among older adults becomes a hot topic in South Korea, as the population of the older adults has been increasing dramatically. The purpose of this study was to explore the longitudinal patterns of the cognitive function and identify factors influencing these changes among Korean older adults without their partner, using latent profile analysis. The samples included 612 adults aged 60 and older without partner from the Korean Longitudinal Study of Aging (KLoSA) in 2016, 2018, and 2020. The logistical regression was used to identify factors influencing group classification. Result revealed four types of the latent profiles were derived based on the cognitive function: the mid-level group, the high-level maintenance group, the low-level maintenance group, and low-level decline group. Factors such as depressive symptoms, social activity participation, and age were significantly associated with cognitive function decline in the high-level maintenance group, and the age was associated with the cognitive function changes. The findings highlight the importance of interventions to prevent cognitive function changes among older adults, considering depressive symptoms, social activity participation, and age.

